# Genetic diversity and population structure of the sweet leaf herb, *Stevia rebaudiana* B., cultivated and landraces germplasm assessed by EST-SSRs genotyping and steviol glycosides phenotyping

**DOI:** 10.1186/s12870-019-2061-y

**Published:** 2019-10-21

**Authors:** Patrick Cosson, Cécile Hastoy, Luis Ernesto Errazzu, Carlos Jorge Budeguer, Philippe Boutié, Dominique Rolin, Valérie Schurdi-Levraud

**Affiliations:** 1 UMR Biologie du Fruit et Pathologie, 1332, INRA Université de Bordeaux, 71 avenue Edouard Bourlaux, 33883 Villenave d’Ornon cedex, France; 2 Oviatis SA, Le Bourg, 47150 Lacaussade, France; 3 INTA INTA-EEA Famaillá CP 4132 Famaillá, Tucumán, Argentina; 4 FAZ, National University of Tucumán, Tucumán, Argentina

**Keywords:** *Stevia rebaudiana*, Genetic diversity, Cultivars, Landraces, Steviol glycosides

## Abstract

**Background:**

*Stevia rebaudiana* (Asteraceae)*,* native from Paraguay, accumulates steviol glycosides (SGs) into its leaves. These compounds exhibit acaloric intense sweet taste which answers to consumer demands for reducing daily sugar intake. Despite the developpement of *S. rebaudiana* cultivation all over the world, the development of new cultivars is very recent, in particular due to a colossal lack of (1) germplasm collection and breeding, (2) studies on genetic diversity and its structuring, (3) genomic tools.

**Results:**

In this study, we developped 18 EST-SSR from 150,258 EST from The Compositae Genome Project of UC Davis (http://compgenomics.ucdavis.edu/data/). We genotyped 145 *S. rebaudiana* individuals, issued from thirty-one cultivars and thirty-one landraces of various origins worldwide. Markers polymorphic information content (PIC) ranged between 0.60 and 0.84. An average of 12 alleles per locus and a high observed heterozygoty of 0.69 could be observed. The landraces revealed twice as many private alleles as cultivars. The genotypes could be clustered into 3 genetic populations. The landraces were grouped in the same cluster in which the oldest cultivars “Eirete” and “MoritaIII” type are also found. The other two clusters only include cultivated genotypes. One of them revealed an original genetic variability. SG phenotypes could not discriminate the three genetic clusters but phenotyping showed a wide range of composition in terms of bitter to sweet SGs.

**Conclusion:**

This is the first study of genetic diversity in *Stevia rebaudiana* involving 145 genotypes, including known cultivars as well as landrace populations of different origin. This study pointed out the structuration of *S. rebaudiana* germplasm and the resource of the landrace populations for genetic improvement, even on the trait of SG’s composition.

## Background

*Stevia rebaudiana,* native from Paraguay, is a perennial species that accumulates steviol glycosides (SGs) into its leaves. These natural compounds exhibit acaloric intense sweet taste. Leaves of *S. rebaudiana* were firstly used as a general sweetening agent by native people of Paraguay and Brazil [1]. Consumer demands for reducing daily sugar intake and for healthy products place *S. rebaudiana* production at the crossroad of these needs. Correlated to growing demand, *S. rebaudiana’s* cultivation is increasing all over the world. The species can be grown in a wide range of climatic areas. Nevertheless, the cultivation of *S. rebaudiana* as a crop is very recent and is done on a small scale in the countries of origin. In 1964, its first commercial cultivation was reported in Paraguay (Katayama et al. 1976; Lewis 1992). Afterwards, great efforts were made by Sumida in 1971 to establish *S. rebaudiana* cultivation in Japan (Crammer & Ikan, 1986). Later on, it was introduced as a crop in many countries. In 2016, 80% of world stevia leaves production was coming from China with 50,000–60,000 tons of dry leaves per year [2]. Other significant producing countries are located in Asia (Indonesia, India, Japan, Korea) and in America (Mexico, USA, and Canada) [3]. Recent regulatory approval explains the newly beginning area of production in Europe [4].

This recently developed crop suffers from a lack of high-value adapted and traceable cultivars. Ninety cultivars have been recently listed by Angelini et al. in 2016 [5]. But, farmers’ practices show that most of the cultivars that are produced are related to cultivars known as “Eirete”, “Criolla” and “Morita” type. They are sold as seeds, often through open-pollinated production as the species is self-incompatible [6]. These genotypes have been mainly bred through mass selection. Besides these currently grown population cultivars, some others are patented as those from the US S&W company [7] or the Malaysian PureCircle. These genotypes are mainly improved for SG’s yield and SG’s composition producing the sweetest taste.

However numerous other traits such as seed germination rate, flowering date, aerial biomass yield, response to biotic and abiotic stresses are poorly improved. Then, identification of productive genetic resources and breeding is clearly needed.

Collecting and studying germplasm is a key pillar to start a breeding program. Diversity in plant genetic resources provides opportunity for plant breeders to develop new and improved cultivars with desirable characteristics and is also fundamental to limit the genetic erosion. No available public collection exists and only private companies own collections that are not available. The phenotypic study of a limited number of genotypes have been conducted in different countries [8–10]. Since many years, the use of molecular markers has improved significantly the management and utilization of crop genetic diversity [11]. In *Stevia*, until recently, the lack of genomic information has made genotyping a bottleneck. Some molecular markers as RAPD [12] and ISSR [13] have been used in previous studies to analyze the diversity in very small collection of individuals. RAPD have also been used to construct the first *S. rebaudiana* genetic map in 1999 [14]. Due to their lack of repeatability these markers were abandoned. Despite the huge development of crop sequencing in the recent years, *S. rebaudiana* has just been sequenced in 2017 by the private consortium PureCircle/Coca-Cola/Keygene [15]. The consortium declared to have sequenced one genotype but sequences have not been released yet. The lack of information on SNP markers confirmed the need to develop SSR markers. For some orphan species as *S. rebaudiana* and for genetic populations analysis SSR remain first choice markers [16, 17]. They still have great applicability due to their high polymorphism, relatively easy scoring, testable neutrality, and Mendelian inheritance. Kaur et al. and Bhandawat et al. [18, 19] developed a set of 52 and 17 SSR markers respectively through the screening of 5548 stevia ESTs sequences from leaf tissues retrieved from the NCBI. These markers were used to classify forty randomly chosen genotypes from selection at CSIR Institute (India) or 12 local genotypes from Northern India. These studies were pioneer in the development and use of SSR markers in *S. rebaudiana*. In 2013, the Compositae Genome Project released EST data from 15 reference transcriptome assemblies for Compositae crops or their wild relatives including *S. rebaudiana* [20]. This large amount of sequences allowed to screen for SSR patterns and develop EST-SSR markers. To our knowledge, in *S. rebaudiana* no studies addressed the question of developing molecular markers to classify germplasm and to question population structure. The purpose of this work was 1) to develop and assess the applicability of EST-SSRs developed for *S. rebaudiana* as markers in thirty-one landraces genotypes and one hundred and fourteen cultivated genotypes issued from thirty-one cultivars; 2) to identify genetic diversity and population structure in *S. rebaudiana*’s landraces and cultivars and 3) to check the link between genetic variability and its structure and phenotypic SG variability in cultivated and landraces populations.

## Results

### EST-SSR genotyping

**Fig. 1:**
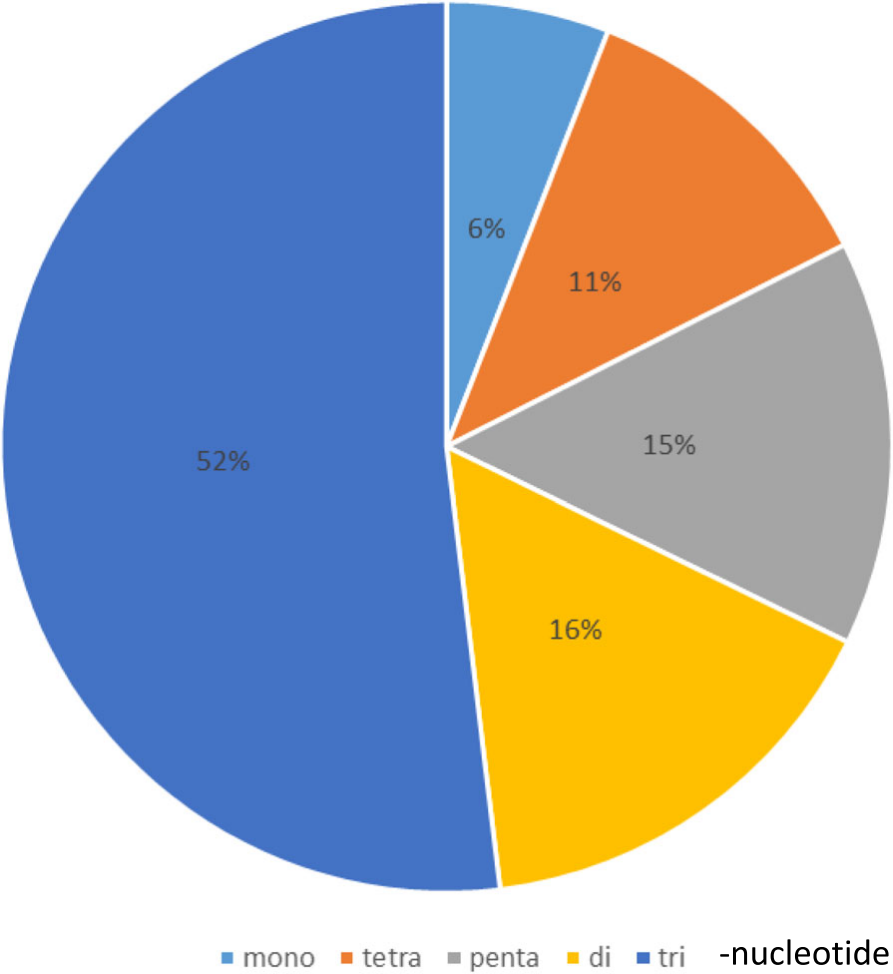
Summary of the distribution of the number of repetitions observed in the total of 1745 unique SSRs selected from the 150,258 unigenes available for *Stevia rebaudiana* at http://compgenomics.ucdavis.edu/data/cwassy_2012/iAssSta.fa (The Compositae Genome Project of UC Davis)

Of the 150,258 unigenes, 3401 SSR pattern could be detected, 1745 being unique. These 1745 SSR are divided into 6% of mono-nucleotide, 16% of di-nucleotide, 52% of tri-nucleotide, 11% of tetra-nucleotide and 15% of penta-nucleotide (Fig. 1 and Additional file 2: Table S1). Among these 1745, 1060 were considered suitable for primer design (Table 1). More than 60% are tri-nucleotide repeat type. Ninety-four with 10 to 26 repeats were selected for primer design (Additional file 1: Figure S1). Screened with 5 genotypes, 19 did not produce any amplification, 5 generated PCR products over 300 bp size and 17 monomorphic or multiloci profiles. Fifty-three primer pairs amplified a polymorphic fragment. Eighteen were selected for population studies, based on the multiplexing possibility (Additional file 2: Table S2).

**Table 1:** Information summary on the number and percentage of each SSR selected through the pipeline Additional file 1: Figure S1

Searching items		Numbers	
Total number of unigene		150,258	
Total number of SSR detected		3401	
Total number of unique SSR (Blast <80%)		1745	
Total number of SSR suitable for primer design ^a^		1060	
	Repeat type		Percentage
	mono-nucleotide	56	5.28
	di-nucleotide	107	10.09
	tri-nucleotide	655	61.79
	tetra-nucleotide	108	10.19
	penta-nucleotide	134	12.64
Total		1060	100.00

^a^ minimum 100 pb each side

### Genetic variability

A panel consisting of 145 individual plants (Table 2 and Additional file 2: Table S3) was genotyped with the 18 selected markers. A total of 213 alleles were detected in the 145 individual plants analyzed (Table 3). The number of alleles detected per locus ranged between 5 and 19 (stvia021; stvia048 respectively) with an average of 12 alleles per locus. Markers’ PICs ranged between 0.60 (stvia021) and 0.84 (stvia025 and stvia051). The average observed and expected heterozygoty across markers were Ho = 0.69 (min–max: 0.46 (stv018)-0.87 (Stv036)) and He = 0.78 (min–max: 0.66–0.86). The allelic richness calculated as the mean number of private alleles ranged from 1.5 for cultivated genotypes to 3 for landraces (Additional file 1: Figure S2).

**Table 2:** List of *Stevia rebaudiana* cultivated and landraces genotypes used in the current study

ID^a^	Type of material^b^	Origin^c^	Number of cultivars^d^	Total number of genotypes
Cult_CAN	Cultivar	Canada	3	13
Cult_CHI	Cultivar	China	2	15
Cult_FRA	Cultivar	France	12	34
Cult_GER	Cultivar	Germany	8	20
Cult_ISR	Cultivar	Israël	1	5
Cult_NET	Cultivar	Netherlands	2	9
Cult_SPA	Cultivar	Spain	3	18
Lr_FOR	Landrace	Argentina		1
Lr_JUJ	Landrace	Argentina		9
Lr_MIS	Landrace	Argentina		9
Lr_TUC	Landrace	Argentina		10
Lr_CUB	Landrace	Cuba		2

^a^Identification

^b^Cultivars refer to sold genotypes through commercial providers. They were provided as seed lots

^c^Country of the provider or origin of the landrace; Landraces from Cuba were provided by the New York Botanical Garden Herbarium, catalog number 1687090 and 1,687,091, collection number 5353, collected in Cuba in 1927 and 1931

^d^Details of ID of each genotype per cultivar is given in Additional file 2: Table S1

**Table 3:** Polymorphism analysis at 18 loci of *Stevia rebaudiana* genome for 145 genotypes

Markers Name	Allele Number	Product size expected	Allele size range	Major Allele Frequency	PIC^a^	Gene Diversity (He)	Heterozygosity (Ho)^b^	Fis W&C^c^
stvia004	17	185	166–226	0.32	0.81	0.83	0.79 ns	0.047
stvia018	13	121	89–134	0.22	0.82	0.84	0.46***	0.452
stvia021	5	119	97–121	0.38	0.60	0.67	0.47***	0.299
stvia024	16	205	173–220	0.25	0.83	0.84	0.69***	0.18
stvia025	15	224	203–243	0.21	0.84	0.86	0.70***	0.193
stvia036	15	163	144–180	0.28	0.83	0.85	0.87***	−0.021
stvia044	9	208	203–221	0.36	0.70	0.74	0.49***	0.341
stvia048	19	153	132–163	0.31	0.82	0.84	0.84 ns	0.002
stvia051	13	194	182–224	0.23	0.84	0.86	0.81***	0.062
stvia057	16	236	190–265	0.28	0.77	0.80	0.68***	0.156
stvia071	8	187	168–191	0.36	0.74	0.77	0.74 ns	0.038
stvia072	10	239	231–251	0.56	0.63	0.65	0.61***	0.057
stvia079	13	173	153–198	0.29	0.80	0.82	0.79***	0.035
stvia084	11	159	142–178	0.49	0.70	0.72	0.70 ns	0.041
stvia093	12	221	205–247	0.38	0.78	0.80	0.79***	0.017
stvia096	8	204	174–213	0.29	0.75	0.78	0.76***	0.033
stvia099	6	111	88–121	0.34	0.66	0.71	0.66 ns	0.076
stvia107	7	125	125–143	0.52	0.61	0.65	0.61 ns	0.062

^a^*PIC* Polymorphic information content

^b^Chi-square test for Hardy-Weinberg equilibrium; *ns* = not significant; ***P < 0.001

^c^*FIS W&C* FIS Weir and Cockerham (1984) [47]

### Population structure

**Fig. 2:**
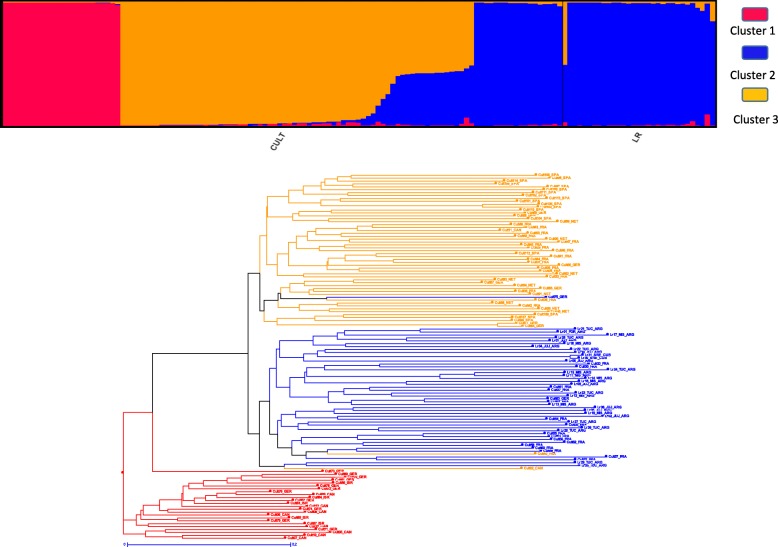
Population structure analysis of the cultivated and landraces stevia inferred using the model-based program STRUCTURE at K = 3. **a** Proportions of ancestry of cultivated and landraces *Stevia rebaudiana* accessions (*n* = 145) inferred with STRUCTURE for K = 3. Each individual is represented by a vertical bar, partitioned into colored segments in proportion of the estimated membership in the different genetic clusters inferred with STRUCTURE. Under the figure are depicted the two groups of genotypes cultivated (CULT) or landraces (LR) and color and names of the three clusters. **b** Neighbor-Joining dendrogram based on DICE dissimilarity indices showing the relationships among the nonadmixed 126 *Stevia rebaudiana* individuals (i.e. individuals assigned to one cluster at K = 3 with a membership coefficient > 0.80). Genotypes were colored according to their assignment to the three different genetic clusters, as inferred by STRUCTURE. Branch length is proportional to the distance between nodes

The change rate in the log-likelihood between successive K values (DK) inferred with STRUCTURE revealed three clusters with a relatively high Δ*K* value at *K* = 3 (Fig. 2a). We used STRUCTURE membership coefficients inferred at K = 3 to define the populations used in subsequent analyses. For analyses hereafter, genotypes were assigned to a given population if their membership coefficient for that population was ≥0.80. Thus, genotypes with an identity value under 80% probability of belonging to a given subpopulation were considered as admixed. Based on this, cluster 1 had 24 accessions, cluster 2 had 48 accessions and cluster 3, 54. Nineteen accessions were admixed (Additional file 2: Table S4). At K = 3 (Fig. 2a), the cultivated Stevia were shown to belong to the three clusters (named cluster 1, 2 and 3) whereas the landraces from Argentina and Cuba belonged to cluster 2. In this cluster 2, cultivated stevia belong to the oldest selections known as “EireteI”, “EireteII” and “MoritaIII” types but also with the “C” and “D” cultivars more recently selected in Germany and belonging to the EUSTAS collection.

### Genetic variation and differentiation among the three stevia clusters

**Fig. 3:**
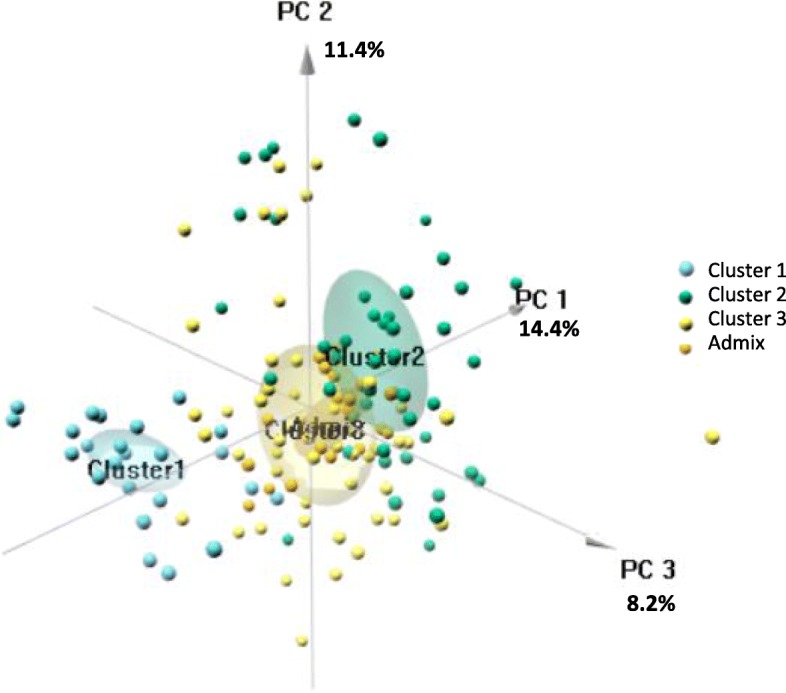
Principal component analysis of the 145 Stevia rebaudiana genotypes for steviol glycosides proportions. Analysis was based on 9 SGs. Graph of variable (left) shows steviol proportion (PST), RebA proportion (PRebA), sweet SG proportion (Psweet; sum of RebM, RebD and RebBF) and bitter SG proportion (Pbitter; sum of RebC, DulA, Rub and SB). Graph of individuals (right) shows the distribution along dimension 1 which explains 95.28% of the variance. The names of the genotypes present at the ends (surrounded in blue) are indicated. The 3 clusters cannot be distinguished (in blue).

We built a Neighbor-Joining tree of the 126 non-admixed individuals (Additional file 2: Table S4) based on dissimilarity scores. The dendrogram showed three major clades (Fig. 2b). Structure of the dendrogram was in agreement with the clustering inferred with STRUCTURE, distinguishing three clades corresponding to clusters 1 (in red), 2 (in blue) and 3 (in orange) except for some genotypes, CULT75_GER, CULT02_CAN and CULT40_FRA. Dendrograms of clusters provided an interesting pattern with a clear differentiation of cultivated stevia cluster 1. The PCA (Fig. 3) revealed a similar pattern as inferred with STRUCTURE, with a clear differentiation of cultivated stevia cluster 1, clearly separated from the other stevia clusters.

We computed population genetic statistics for the three genetic populations (Table 4). The inbreeding coefficients (Fis) were all low but positive. This result can be linked to the significant Chi-square test for Hardy-Weinberg equilibrium on most of the markers (Table 3), related to the putative kinship within the seeds lots. High genetic diversity was found in the cultivated populations clusters 1 and 3 with He = 0.725 and 0.753 respectively and in the mixed landraces and cultivated cluster 2 (He = 0.801).

**Table 4:** Summary statistics of genetic variation among the three *Stevia rebaudiana* populations detected with STRUCTURE

Pop		N	Na	Ne	Ho	He	Fis
Cluster1	Mean	24	6.444	3.833	0.623	0.725	0.140
	SE		0.372	0.203	0.040	0.016	0.054
Cluster2	Mean	48	10.778	5.626	0.723	0.801	0.100
	SE		0.823	0.433	0.031	0.017	0.031
Cluster3	Mean	54	8.611	4.441	0.690	0.753	0.083
	SE		0.652	0.307	0.031	0.020	0.032

N = sample size

Na = No. of Different Alleles

Ne = No. of Effective Alleles = 1/(Sum p_i_^2^)

Ho = Observed Heterozygosity = No. of Heterozygotes/N

He = Expected Heterozygosity = 1 - Sum p_i_^2^

He = Unbiased Expected Heterozygosity = (2 N / (2 N-1)) * He

Fis = Fixation Index = (He - Ho) / He = 1 - (Ho / He)

Where p_i_ is the frequency of the i^th^ allele for the population & Sum p_i_^2^ is the sum of the squared population allele frequencies

Pairwise FST among the three stevia populations were all significant (Table 5). They indicated a differentiation between the three populations. AMOVA was used to estimate the variance among and within populations (Table 6). The results indicated that the majority of genetic variation was due to a remarkable degree of within population variation (98%; Table 6). Only 2% of the genetic variation was attributed to differences among populations.

**Table 5:** Pairwise Population Matrix of Fst Values for Total

	Cluster1	Cluster2	Cluster3
Cluster1	0.000	0.001	0.046
Cluster2	0.020	0.000	0.004
Cluster3	0.013	0.012	0.000

Fst values below the diagonal. Probability, *P*(rand > = data) based on 999 permutations is shown above diagonal

**Table 6:** Analysis of molecular variance (AMOVA) using SSR data for the three subpopulations of *S. rebaudiana*

Souce of variation	d.f.	Sum of squares	Variance components	Percentage of variation
Among populations	2	31.395	0.10901	1.54
Within populations	249	1737.256	6.97693	98.46
Total	251	1768.651	7.08594	

### Variation of steviol glycosides composition among Stevia populations

**Fig. 4:**
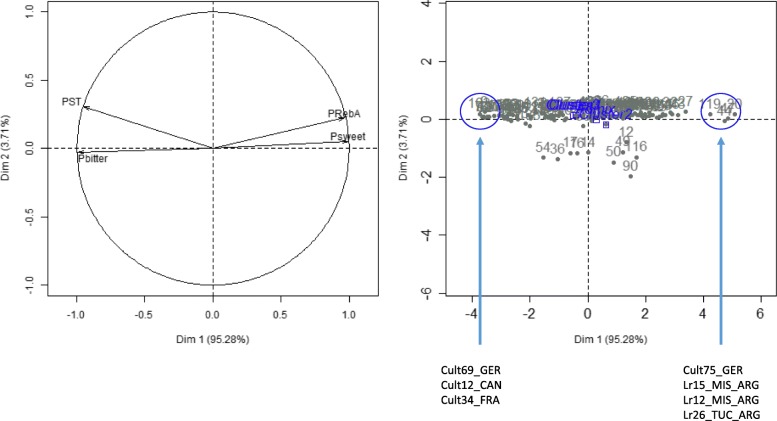
Principal component analysis of the 145 *Stevia rebaudiana* genotypes for steviol glycosides proportions. Analysis was based on 9 SGs. Graph of variable (left) shows steviol proportion (PST), RebA proportion (PRebA), sweet SG proportion (Psweet; sum of RebM, RebD and RebBF) and bitter SG proportion (Pbitter; sum of RebC, DulA, Rub and SB). Graph of individuals (right) shows shows the distribution along dimension 1 which explains 95.28% of the variance. The names of the genotypes present at the ends (surrounded in blue) are indicated. The 3 clusters cannot be distinguished (in blue)

The analyses above revealed high genetic diversity in cultivated as in stevia landraces. In order to evaluate the classification of the different populations according to their SG composition, we estimated the ratio of steviol (ST), RebA, sweet SG as the sum of RebM, RebD and RebBF and bitter SG as the sum of RebC, DulA, Rub and SB. Expectedly, the graph of the variables contrasted the sweet-flavored SGs such as RebA and sweet SG with stevioside and bitter SG (Fig. 4). The graph of the individuals showed a distribution along the first dimension separating the genotypes presenting the most sweetie taste SG until those presenting a dominant bitterness. Interestingly, the 3 genetic populations appear little differentiated for the content trait in the different SGs, the 3 clusters being grouped in the center of the graph of the individuals. Clusters 1 and 3 appear superimposed. Nevertheless, it should be noted that the cluster 2, stands out slightly towards the highest compositions in SG of sweet taste. Thus, the genotypes that explain the most the composition of sweet taste SG are 3 genotypes of populations of Argentina (Lr15_MIS_ARG, Lr12_MIS_ARG and Lr26_TUC_ARG) as well as the cultivated genotype Cult75_GER which is the improved genotype “C” of the EUSTAS collection (Hastoy et al., 2019). The genotypes that explain the most the composition in bitter SG are essentially cultivated genotypes such as Cult69_GER, Cult12_CAN and Cult34_FRA.

## Discussion

The gathering and in-depth study of genetic resources is an essential step in setting up a breeding program. *S. rebaudiana* has a recognized interest in health and the associated industry is now highly developed and world-wide. Nevertheless, the optimized improvement of Stevia is in its very early stages. Breeding programs are relatively new. In particular, they suffer from the critical lack of in-depth information on genetic resources associated with a lack of genomic tools. On the basis of the gathering of 145 genotypes of origin as diverse as possible, our study has for objective (1) to develop robust SSR markers (2) to study the pattern of genetic diversity and genetic structure of 114 cultivated genotypes from 31 cultivars but also 31 landraces coming from different regions of Argentina and Cuba.

### SSR polymorphism

SSR markers are useful for studying genetic diversity because they are highly polymorphic, multi-allelic and their development requires a limited genomic information. Therefore, they are the easiest and more informative molecular markers. The development of molecular markers in *S. rebaudiana* is longstanding. In 1999, one hundred and eighty-three RAPD markers were developed and used to produce the first partial genetic map from a pseudo test-cross F1 [14]. RAPD and ISSR markers were then used in three studies to study the relationships between 6 Egyptian and Indonesian accessions in 2008 and 2011 respectively [13, 21] and 12 accessions from India in 2016 [22]. As underlined in [19], the dominance and low reproducibility rate of such markers lead to the development of EST-SSR markers. EST-SSR were developed from 2977 unigenes predicted from 5548 publicly available ESTs of *S. rebaudiana* by [18, 19]. Fifty-two and seventeen EST-SSR were developed respectively. They were used to study the genetic variability of forty randomly genotypes from CSIR, India [19] and twelve accessions from North India [18, 22]. These works reported a number of alleles per locus ranging between 2 and 15, with an average of 4.7 allele per SSR locus which is lower than what we found. This could be explained by the areas of the genome targeted by SSRs and/or by a narrower genetic diversity in Bhandawat et al. studies [19] and in Kaur et al. studies [18], focused on Indian genotypes. It has also been shown in durum wheat [23], in eggplant [24] or in opium poppy [25] that genomic SSRs are more polymorphic than EST-SSRs.

Nevertheless, in both studies and our, average Ho was high, 0.80 and 0.69 respectively, in accordance with the reported outcross mating system of *S. rebaudiana* [6].

### Population structure and genetic diversity

The population structure and dendogram analyses divided the accessions into three clusters/subpopulations, although the analyses are based on different methods. The low number of admixed genotypes (19/145) can be explained by the composition of the collection, 78% (114/145) of which are cultivated varieties and 22% (31/145) of landraces. No wild genotypes could be analyzed. In addition, the varieties grown are often produced by crossing between two identified parents. The use of a limited number of parents may also explain the low number of admixed.

Except for the two Chinese cultivars belonging to admix cluster, no regional aggregation could be observed. Cluster 1, in particular, gathers genotypes from Canada, Germany and Israël, indicating large seed and alleles exchanges in Stevia. Cluster 1 and Cluster 3 consisted essentially of cultivated varieties. Cluster 1 seems to reveal original diversity. It contains the genotype “Gawi” selected in Germany [26, 27] but of unknown origin [28]. Nevertheless, in our previous study on the phenotype of 15 genotypes from various origin in southwestern France field conditions, “Gawi’ was shown to belong to a different morphotype from “Eirete” on the basis of foliar biomass production [28]. Cluster 3 also consist of cultivated varieties, some of them being sold as “Criolla” type. In a very interesting way, the Argentine landraces as well as the old samples from Cuba are all grouped in the cluster 2. Landraces revealed a mean number of private allele per locus around twice the mean of cultivated genotypes. This cluster is shared with cultivated varieties, type “Eirete” and “MoritaIII”, known to be varieties formerly improved and very widespread in the world.

AMOVA results showed that the genetic differentiation was overwhelmingly due to within population variation. As our study is the first large study on *S. rebaudiana* genetic resources, there is no reference study. But, similar results have been observed in other outcrossing perennials such as alfalfa (*Medicago sativa*) [29], Dalmatian Pyrethrum (*Tanacetum cinerariifolium*) [30] or agronomic switchgrass (*Panicum virgatum*) [31]. The high level of genetic variability observed within populations of such species is most likely due to their partial or completely allogamous reproductive systems.

### SG phenotyping

Phenotyping of SGs does not allow to structure the different populations. The three genetic populations differed very little based on this trait. Surprisingly, it is the landraces populations that appear to draw towards the quality of sweetness in SG, while cluster 1 and 3, composed only of cultivars cannot be distinguished. They are composed of cultivars producing very varied SGs, from bitter to sweet, which may seem surprising considering that the ratio SG soft/bitter is the first selection criterion in Stevia [5].

## Conclusions

Our study is the first analysis of genetic diversity in *S. rebaudiana* involving 145 genotypes, including known cultivars as well as landrace populations of different origin. This study generated 18 new highly polymorphic and robust microsatellite markers. These markers are of great potential for genetic diversity evaluation and germplasm managing which is a crucial step of breeding. These markers could also be used for genotypes traceability. The 145 genotypes of *S. rebaudiana* were successfully genotyped. They revealed three genetic populations with a remarkable variation within population. Landraces revealed their allelic richness and their interest in term of sweet SG phenotype. They harbor valuable genetic variation for further improvement via breeding as shown through their phenotyping in Argentina [32].

## Methods

### Plant materials

A panel consisting of 145 individual plants belonging to *S. rebaudiana* was collected from different origins (Table 2 and Additional file 2: Table S3). One hundred and fourteen genotypes called “cultivated” issued from thirty-one cultivars were obtained from different providers as seed lots and distributed as follow: 13 from Canada, 15 from China, 34 from France, 20 from Germany, 5 from Israël, 9 from The Netherlands and 18 from Spain. Thirty-one are landraces. Twenty-nine are originated from North Argentina, regions of Formosa, Tucuman, Jujuy and Misiones and belong to INTA collection [32]. Two, catalog number 1687090 and 1,687,091, collection number 5353, were provided by the New York Botanical Garden Herbarium. They were collected in Cuba in 1927 and 1931, respectively.

### SSR genotyping

#### Identification of EST-SSR markers

Microsatellite or SSRs were developed from 150,258 EST generated on 454 sequencing and downloaded from The Compositae Genome Project of UC Davis (http://compgenomics.ucdavis.edu/data/cwassy_2012/iAssSta.fa) according to the pipeline described in Additional file 1: Figure S1. The program Sputnik (http://abajian.net/sputnik/) were used to identify EST containing SSR. For SSR identification, the minimum motif repeats were defined as 20 repeats for a mononucleotide unit, 10 repeats for a dinucleotide unit, 7 repeats for trinucleotide unit, 5 repeats for tetranucleotide unit and 4 repeats for pentanucleotide unit. Primer pairs flanking the SSRs were designed using Primer3Plus [33] with melting temperatures 52–57 °C, primer lengths 18–24 bp, expected fragment size 100–300 bp. All the designed primers were screened on five samples, Cult32_FRA, Cult33_FRA, Cult34_FRA, Cult36_FRA, Cult37_FRA. The primers producing clear and polymorphic bands were subsequently used for genetic diversity assessments.

#### DNA extraction and molecular genotyping

Genomic DNA was extracted using a modified previously published protocol [34]. Young leaves were dehydrated in the oven at 55 °C for 48 h, and then ground to fine powder in a Grinobender. One ml of buffer (0.1 M Tris-HCl pH 8, 0.7 M NaCl, 0.04 M EDTA, 1% HATMAB, plus 1% β-mercaptoethanol and 50 μg/ml proteinase K added just before use) was added to 30 mg of powder and incubated for 60 min at 65 °C, gently mixing by inversion every 15 min. After Chloroform: IsoAmylic Alcohol extraction, isopropanol precipitation and 70% ethanol washing, DNA was resuspended in 100 μl of pure water. Genomic DNA was quantified on Epoch Microplate Spectrophotometer (BioTek).

PCR amplifications were performed in 15 μl reaction volume containing 10 ng of template DNA, 1 X PCR buffer (10 mM Tris-HCl pH 8.3; 50 mM KCl), 2.5 mM MgCL_2_, 0.2 μM of each primer, 200 μM of each DNTP’s and 0.5 U SurePRIME™ DNA polymerase (MP Biomedicals). The amplifications were performed in a Mastercycler Pro (Eppendorf) with the following PCR protocol: 15 min initial denaturation at 95 °C and 35 cycles of 30 s at 94 °C, 45 s at 55 °C, 60 s at 72 °C, followed by a final extension for 10 min at 72 °C.

PCR amplicons were separated on denaturing polyacrylamide gels consisting of 4.5% polyacrylamide (acrylamide: bis-acrylamide 19: 1) and 7 M urea in 1× TBE buffer. After run, amplified fragments were visualized using silver staining protocol [35].

Fragments were genotyped at each locus, and the allele sizes were scored using 10–330 bp ladder (Invitrogen).

### Genetic variation

Allele frequencies and genetic diversity measures were calculated using PowerMarker 3.25 [36] and GenAlEx 6.5 [37]. These measures included number of alleles (Na), major allele frequency, polymorphic information content (PIC), expected heterozygosity or Gene Diversity (He), observed heterozygosity (Ho). Inbreeding coefficient (Fis) was calculated using SPAGEDI 1.3 [38] and verified with GENETIX v4.05 [39]. Private allelic richness was computed with ADZE software to adjust for sample size differences [40]. We further explored the genetic differentiation and relationships among samples using an unweighted Neighbor-Joining tree constructed using simple matching dissimilarity indices of Jaccard’s coefficient method and bootstrap values over 2000 replicates as implemented in the DARWIN software package v6.0.010 [41]. Among-population FST and Nei’s indices were estimated using ARLEQUIN v3.5 [42]. The significance of FST was assessed by random resampling of the genotypic data through 1000 permutations.

### Population structure

Representation of the genetic relationships among individuals was explored with a principal component analysis (PCA) performed with GENETIX v4.05 [39]. We also used the individual based Bayesian clustering method implemented in STRUCTURE 2.3.3 [43] to investigate population subdivision. We ran STRUCTURE from K = 2 to K = 10 using admixture and correlated allele frequencies assuming no prior population information. Burn-in and number of Markov chain Monte Carlo iterations were set to 10,000 and 100,000, respectively. Ten independent runs were carried out for each K, and outputs were processed with CLUMPP V1.1.2 [44]. STRUCTURE barplots were displayed using DISTRUCT 1.1 [45]. We examined the distribution of ΔK, plotted with STRUCTURE harvester (http://taylor0.biology.ucla.edu/structureHarvester/) according to Evanno et al. (2005) [46].

### Steviol glycosides extraction and quantification

Steviol glycosides extraction and quantification was done as described in Hastoy et al. (2019) [28]. Nine SGs were detected at 202 nm (RebD, RebM, ST, RebA, RebC, DulA, Rub, RebB, SB) and previously identified by purified SG standard (Chromadex, USA). For each SG, a standard range between 5 and 1000 ng/μL of purified standard was used to quantify each amount. Results were expressed as content per unit of leaf dry weight (% w/w) for each SG and total SGs, and as a proportion (%) of the content of each SG to total SG content.

## Supplementary information

**Additional file 1: Figure S1.** Summary of the pipeline for the selection of the 18 SSRs used in the current study. **Figure S2.** Mean number of private alleles per locus and mean number of distinct allele per locus for the cultivated and landraces genotypes computed with ADZE software

**Additional file 2: Table S1.** Type and number of repeat pattern of the 1745 unique SSR among the 150,258 unigenes available for *Stevia rebaudiana* at http://compgenomics.ucdavis.edu/data/cwassy_2012/iAssSta.fa (The Compositae Genome Project of UC Davis). **Table S2.** List of the 18 SSR and related primers and characteristics used in this study. **Table S3.** List of *Stevia rebaudiana* cultivated and landraces groups studied. **Table S4.** Distribution of the studied genotypes in the different clusters and admix following the analysis by Structure

## Data Availability

All data generated or analysed during this study are included in this published article as supplementary information files.
