# Supplementary material for: Genetic diversity and population structure of the sweet leaf herb, Stevia rebaudiana B., cultivated and landraces germplasm assessed by EST-SSRs genotyping and steviol glycosides phenotyping
Source: BMC Plant Biol. 2019 Oct 21;19:436. doi: 10.1186/s12870-019-2061-y (PMC6805397; doi:10.1186/s12870-019-2061-y)
Supplement: Supplementary file 1 — Additional file 1: Figure S1. Summary of the pipeline for the selection of the 18 SSRs used in the current study. Figure S2. Mean number of private alleles per locus and mean number of distinct allele per locus for the cultivated and landraces genotypes computed with ADZE software [file 12870_2019_2061_MOESM1_ESM.pptx]

## Slide 1
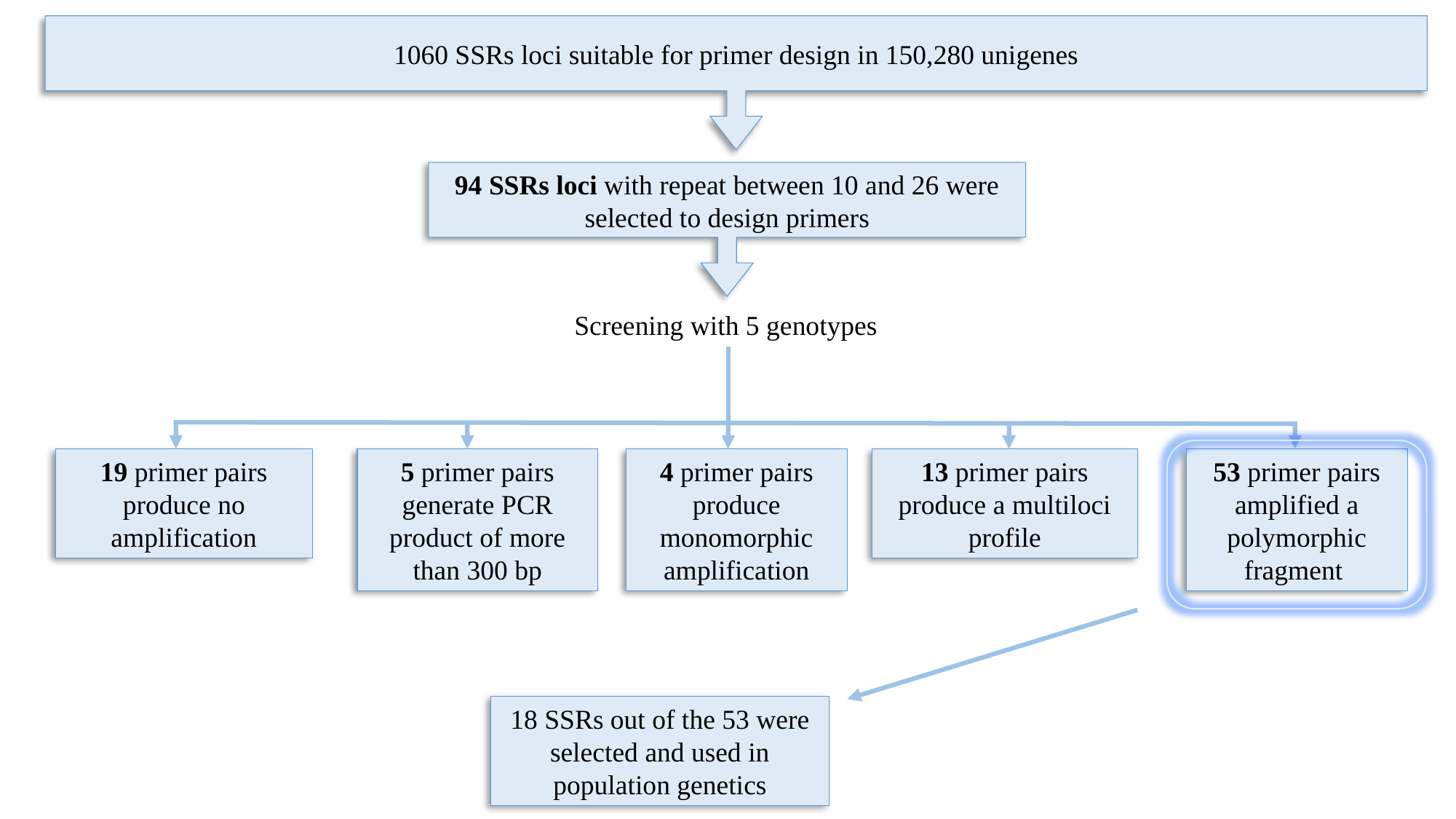

1060 SSRs loci suitable for primer design in 150,280 unigenes
94 SSRs loci with repeat between 10 and 26 were selected to design primers
Screening with 5 genotypes
19 primer pairs produce no amplification
5 primer pairs generate PCR product of more than 300 bp
4 primer pairs produce monomorphic amplification
13 primer pairs produce a multiloci profile
53 primer pairs amplified a polymorphic fragment
18 SSRs out of the 53 were selected and used in population genetics

## Slide 2
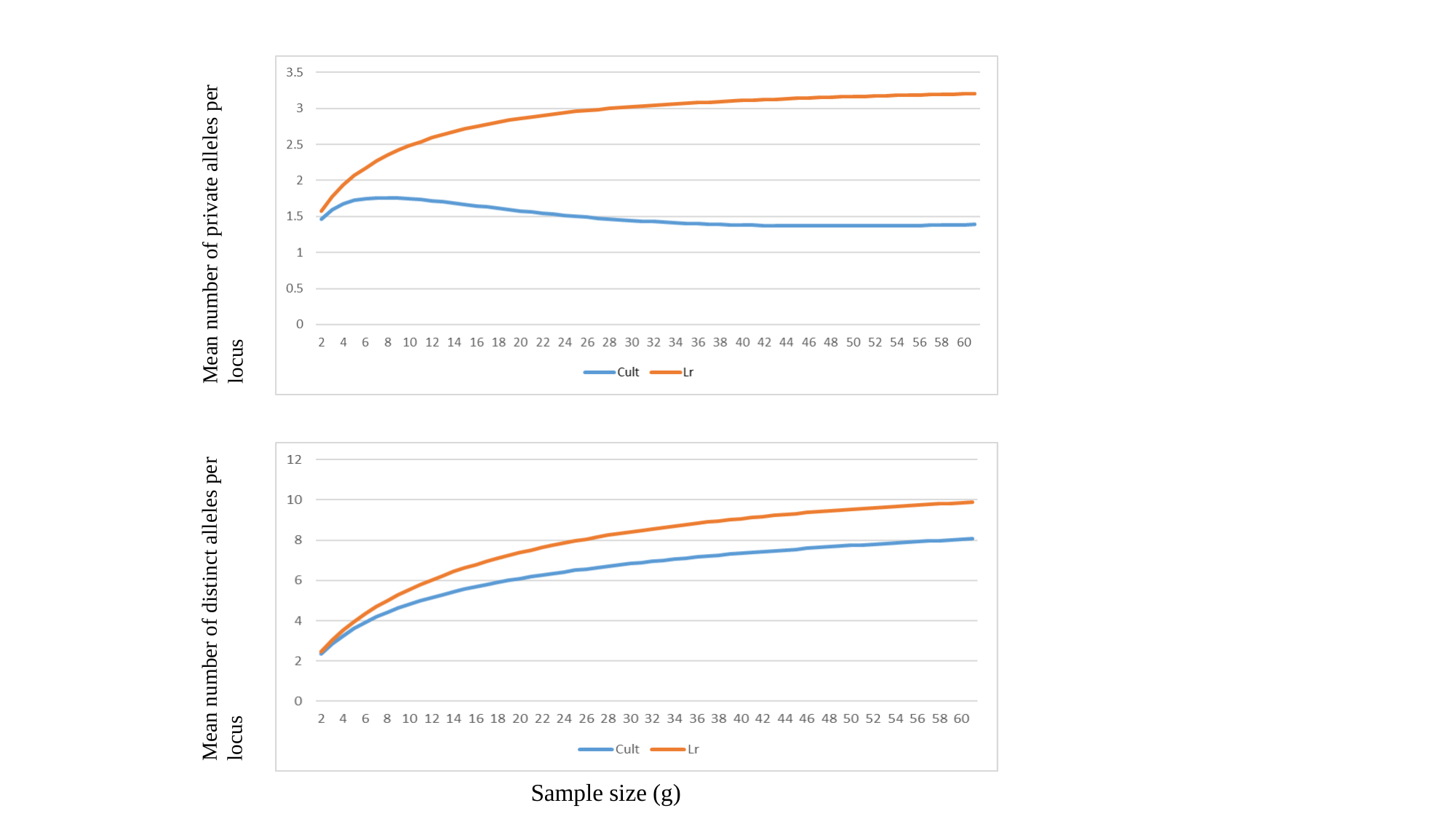

Mean number of private alleles per locus
Mean number of distinct alleles per locus
Sample size (g)
