# Supplementary material for: Genetic diversity and population structure of the sweet leaf herb, Stevia rebaudiana B., cultivated and landraces germplasm assessed by EST-SSRs genotyping and steviol glycosides phenotyping
Source: BMC Plant Biol. 2019 Oct 21;19:436. doi: 10.1186/s12870-019-2061-y (PMC6805397; doi:10.1186/s12870-019-2061-y)
Supplement: Supplementary file 2 — Additional file 2: Table S1. Type and number of repeat pattern of the 1745 unique SSR among the 150,258 unigenes available for Stevia rebaudiana at http://compgenomics.ucdavis.edu/data/cwassy_2012/iAssSta.fa (The Compositae Genome Project of UC Davis). Table S2. List of the 18 SSR and related primers and characteristics used in this study. Table S3. List of Stevia rebaudiana cultivated and landraces groups studied. Table S4. Distribution of the studied genotypes in the different clusters and admix following the analysis by Structure [file 12870_2019_2061_MOESM2_ESM.docx]

Table S1: Type and number of repeat pattern of the 1745 unique SSR among the 150,258 unigenes available for *Stevia rebaudiana* at <http://compgenomics.ucdavis.edu/data/cwassy_2012/iAssSta.fa> (The Compositae Genome Project of UC Davis)

| **Repeats** | **Total** |
| --- | --- |
| A/T | 92 |
| C/G | 11 |
| AC/GT | 66 |
| AG/CT | 101 |
| AT/AT | 110 |
| AAC/GTT | 39 |
| AAG/CTT | 84 |
| AAT/ATT | 218 |
| ACC/GGT | 91 |
| ACG/CGT | 7 |
| ACT/AGT | 34 |
| AGC/GCT | 11 |
| AGG/CCT | 2 |
| ATC/GAT | 417 |
| CCG/CGG | 3 |
| AAAC/GTTT | 12 |
| AAAG/CTTT | 13 |
| AAAT/ATTT | 77 |
| AACC/GGTT | 5 |
| AACG/CGTT | 2 |
| AACT/AGTT | 15 |
| AAGG/CCTT | 5 |
| AAGT/ACTT | 7 |
| AATC/GATT | 8 |
| AATG/CATT | 2 |
| AATT/AATT | 13 |
| ACAT/ATGT | 13 |
| ACCC/GGGT | 3 |
| ACCT/AGGT | 3 |
| ACGT/ACGT | 8 |
| ACTC/GAGT | 1 |
| AGAT/ATCT | 3 |
| AGGG/CCCT | 1 |
| ATCC/GGAT | 2 |
| ATCG/CGAT | 8 |
| CCCG/CGGG | 1 |
| CCGG/CCGG | 1 |
| AAAAC/GTTTT | 24 |
| AAAAG/CTTTT | 12 |
| AAAAT/ATTTT | 43 |
| AAACC/GGTTT | 11 |
| AAACG/CGTTT | 5 |
| AAACT/AGTTT | 11 |
| AAAGC/GCTTT | 1 |
| AAAGG/CCTTT | 7 |
| AAAGT/ACTTT | 4 |
| AAATC/GATTT | 10 |
| AAATG/CATTT | 2 |
| AAATT/AATTT | 8 |
| AACAC/GTGTT | 5 |
| AACAG/CTGTT | 1 |
| AACAT/ATGTT | 2 |
| AACCC/GGGTT | 9 |
| AACCG/CGGTT | 5 |
| AACCT/AGGTT | 6 |
| AACGG/CCGTT | 1 |
| AACGT/ACGTT | 11 |
| AACTT/AAGTT | 9 |
| AAGAG/CTCTT | 2 |
| AAGAT/ATCTT | 1 |
| AAGCT/AGCTT | 1 |
| AAGGT/ACCTT | 3 |
| AATAC/GTATT | 1 |
| AATAG/CTATT | 1 |
| AATAT/ATATT | 11 |
| AATCC/GGATT | 5 |
| AATCG/CGATT | 2 |
| AATCT/AGATT | 2 |
| AATGG/CCATT | 1 |
| AATGT/ACATT | 2 |
| AATTC/GAATT | 3 |
| ACATG/CATGT | 1 |
| ACCAT/ATGGT | 4 |
| ACCCG/CGGGT | 3 |
| ACCCT/AGGGT | 1 |
| ACCGT/ACGGT | 5 |
| ACCTC/GAGGT | 1 |
| ACCTG/CAGGT | 2 |
| ACTAG/CTAGT | 1 |
| ACTAT/ATAGT | 3 |
| AGAGC/GCTCT | 1 |
| AGAGG/CCTCT | 1 |
| AGATC/GATCT | 2 |
| AGATG/CATCT | 3 |
| AGGAT/ATCCT | 1 |
| ATATC/GATAT | 4 |
| ATCCG/CGGAT | 1 |

Table S2: List of the 18 SSR and related primers and characteristics used in this study

| **Markers** | **Forward primer (5'-3')** | **Reverse primer (5'-3')** | **Repeat motif** | **Target UN** |
| --- | --- | --- | --- | --- |
| stvia004 | CTAACCCTCAATTCCTACATCC | CACGTCACTTGCATTTTCC | (AC)19 | UN123762 |
| stvia018 | TTACTAGAGGTAGCATAATTTTCG | GCACATCTACCATATCTAATGC | (ATC)15 | UN037591 |
| stvia021 | AGCTCCAGATAAACAACAGC | ATCTGATCTGTGCATTTTCC | (ATAG)8 | UN149594 |
| stvia024 | AAACCACCCAAGAAATCC | TGTCGGAGTATCAACAGTACC | (AAT)14 | UN147565 |
| stvia025 | TAGTTTGGCCCATTTGACC | AGAGCAGACACCAGATGAGG | (TG)15 | UN023398 |
| stvia036 | TGTCTCTGACAAAATTTATACGG | TTGTCTGTCACCCTGTGG | (AG)13 | UN055377 |
| stvia044 | TGATAGTATAAGGCTTCCGTACC | ACACACCGACGAGACTCC | (AG)13 | UN059205 |
| stvia048 | ATGCCTATCCTTGTGTTGC | TTCAGGTCATTGTGACTGG | (GA)13 | UN045654 |
| stvia051 | AGAAGCTGGAAGAATTGAGC | AATCTCAACCAAACAGAGACG | (TGA)12 | UN028295 |
| stvia057 | ACATTTAGTGAGTGAGTGATTACC | TCTTGGTTTACTGGTGAGG | (TCA)11 | UN025483 |
| stvia071 | TACTAGGATTCTTGATTTGGTGTT | TCAACCACCCGAAACTTTA | (CA)13 | UN031209 |
| stvia072 | TATCTTCTTCAGGAGTCCAAGC | AGATTATTGGCTTCCATTGC | (AC)12 | UN006985 |
| stvia079 | GAACTCAAAGAGGCGATTTC | TCAAATAACTGTCAAAGATGGTG | (ATT)11 | UN092146 |
| stvia084 | TTTGTTCTCCACATGAAACG | ATTGCATCTCTCTCAACTTCAAC | (TAT)11 | UN098000 |
| stvia093 | GAGCTTCCACCATTCTCAGTA | TACAACGAGCATCCCATTC | (ATC)33 | UN016158 |
| stvia096 | AATCTTCAAGTGTCATTTCACC | AAGACGGTTGATGAAGTATGG | (ATC)19 | UN072161 |
| stvia099 | TAAAGATTGCATCAACCGATC | CAAGTTATCCGTCAGTATTCGAT | (AAT)14 | UN104660 |
| stvia107 | TCTGTGGCAAATAACTCTGC | AAGAATCCATTGGCTGATAAC | (AT)13 | UN146828 |

Table S3: List of *Stevia rebaudiana* cultivated and landraces groups studied

| ID^a^ | Type of material^b^ | Origin^c^ |
| --- | --- | --- |
| Cult01_CAN | Cultivar 1 | Canada |
| Cult02_CAN | Cultivar 1 | Canada |
| Cult03_CAN | Cultivar 1 | Canada |
| Cult04_CAN | Cultivar 1 | Canada |
| Cult05_CAN | Cultivar 2 | Canada |
| Cult06_CAN | Cultivar 2 | Canada |
| Cult07_CAN | Cultivar 2 | Canada |
| Cult08_CAN | Cultivar 2 | Canada |
| Cult09_CAN | Cultivar 2 | Canada |
| Cult10_CAN | Cultivar 3 | Canada |
| Cult11_CAN | Cultivar 3 | Canada |
| Cult12_CAN | Cultivar 3 | Canada |
| Cult13_CAN | Cultivar 3 | Canada |
| Cult14_CHI | Cultivar 1 | China |
| Cult15_CHI | Cultivar 1 | China |
| Cult16_CHI | Cultivar 1 | China |
| Cult17_CHI | Cultivar 1 | China |
| Cult18_CHI | Cultivar 1 | China |
| Cult19_CHI | Cultivar 1 | China |
| Cult20_CHI | Cultivar 1 | China |
| Cult21_CHI | Cultivar 1 | China |
| Cult22_CHI | Cultivar 2 | China |
| Cult23_CHI | Cultivar 2 | China |
| Cult24_CHI | Cultivar 2 | China |
| Cult25_CHI | Cultivar 2 | China |
| Cult26_CHI | Cultivar 2 | China |
| Cult27_CHI | Cultivar 2 | China |
| Cult28_CHI | Cultivar 2 | China |
| Cult29_FRA | Cultivar 1 | France |
| Cult30_FRA | Cultivar 2 | France |
| Cult31_FRA | Cultivar 3 | France |
| Cult32_FRA | Cultivar 4 | France |
| Cult33_FRA | Cultivar 5 | France |
| Cult34_FRA | Cultivar 5 | France |
| Cult35_FRA | Cultivar 5 | France |
| Cult36_FRA | Cultivar 6 | France |
| Cult37_FRA | Cultivar 7 | France |
| Cult38_FRA | Cultivar 5 | France |
| Cult39_FRA | Cultivar 5 | France |
| Cult40_FRA | Cultivar 5 | France |
| Cult41_FRA | Cultivar 5 | France |
| Cult42_FRA | Cultivar 8 | France |
| Cult43_FRA | Cultivar 5 | France |
| Cult44_FRA | Cultivar 5 | France |
| Cult45_FRA | Cultivar 5 | France |
| Cult46_FRA | Cultivar 9 | France |
| Cult47_FRA | Cultivar 9 | France |
| Cult48_FRA | Cultivar 9 | France |
| Cult49_FRA | Cultivar 9 | France |
| Cult50_FRA | Cultivar 9 | France |
| Cult51_FRA^d^ | Cultivar 10 | France |
| Cult52_FRA^d^ | Cultivar 10 | France |
| Cult53_FRA^d^ | Cultivar 10 | France |
| Cult54_FRA^d^ | Cultivar 10 | France |
| Cult55_FRA^d^ | Cultivar 10 | France |
| Cult56_FRA^d^ | Cultivar 10 | France |
| Cult57_FRA^d^ | Cultivar 10 | France |
| Cult58_FRA^d^ | Cultivar 11 | France |
| Cult59_FRA^e^ | Cultivar 12 | France |
| Cult60_FRA^e^ | Cultivar 12 | France |
| Cult61_FRA^e^ | Cultivar 12 | France |
| Cult62_FRA^e^ | Cultivar 12 | France |
| Cult63_GER^f^ | Cultivar 1 | Germany |
| Cult64_GER | Cultivar 2 | Germany |
| Cult65_GER | Cultivar 3 | Germany |
| Cult66_GER | Cultivar 3 | Germany |
| Cult67_GER | Cultivar 3 | Germany |
| Cult68_GER | Cultivar 3 | Germany |
| Cult69_GER | Cultivar 3 | Germany |
| Cult70_GER | Cultivar 4 | Germany |
| Cult71_GER | Cultivar 4 | Germany |
| Cult72_GER | Cultivar 4 | Germany |
| Cult73_GER | Cultivar 4 | Germany |
| Cult74_GER | Cultivar 4 | Germany |
| Cult75_GER^g^ | Cultivar 5 | Germany |
| Cult76_GER^h^ | Cultivar 6 | Germany |
| Cult77_GER | Cultivar 7 | Germany |
| Cult78_GER | Cultivar 8 | Germany |
| Cult79_GER | Cultivar 8 | Germany |
| Cult80_GER | Cultivar 8 | Germany |
| Cult81_GER | Cultivar 8 | Germany |
| Cult82_GER | Cultivar 8 | Germany |
| Cult83_ISR | Cultivar 1 | Israël |
| Cult84_ISR | Cultivar 1 | Israël |
| Cult85_ISR | Cultivar 1 | Israël |
| Cult86_ISR | Cultivar 1 | Israël |
| Cult87_ISR | Cultivar 1 | Israël |
| Cult88_NET | Cultivar 1 | Netherlands |
| Cult89_NET | Cultivar 1 | Netherlands |
| Cult90_NET | Cultivar 1 | Netherlands |
| Cult91_NET | Cultivar 1 | Netherlands |
| Cult92_NET | Cultivar 1 | Netherlands |
| Cult93_NET | Cultivar 2 | Netherlands |
| Cult94_NET | Cultivar 2 | Netherlands |
| Cult95_NET | Cultivar 2 | Netherlands |
| Cult96_NET | Cultivar 2 | Netherlands |
| Cult97_SPA^i^ | Cultivar 1 | Spain |
| Cult98_SPA^i^ | Cultivar 1 | Spain |
| Cult99_SPA^i^ | Cultivar 1 | Spain |
| Cult100_SPA^i^ | Cultivar 1 | Spain |
| Cult101_SPA^i^ | Cultivar 1 | Spain |
| Cult102_SPA | Cultivar 2 | Spain |
| Cult103_SPA | Cultivar 2 | Spain |
| Cult104_SPA | Cultivar 2 | Spain |
| Cult105_SPA | Cultivar 3 | Spain |
| Cult106_SPA | Cultivar 3 | Spain |
| Cult107_SPA | Cultivar 3 | Spain |
| Cult108_SPA | Cultivar 3 | Spain |
| Cult109_SPA | Cultivar 3 | Spain |
| Cult110_SPA | Cultivar 3 | Spain |
| Cult111_SPA | Cultivar 3 | Spain |
| Cult112_SPA | Cultivar 3 | Spain |
| Cult113_SPA | Cultivar 3 | Spain |
| Cult114_SPA | Cultivar 3 | Spain |
| Lr01_FOR_ARG | Landrace^j^ | Argentina |
| Lr02_JUJ_ARG | Landrace | Argentina |
| Lr03_JUJ_ARG | Landrace | Argentina |
| Lr04_JUJ_ARG | Landrace | Argentina |
| Lr05_JUJ_ARG | Landrace | Argentina |
| Lr06_JUJ_ARG | Landrace | Argentina |
| Lr07_JUJ_ARG | Landrace | Argentina |
| Lr08_JUJ_ARG | Landrace | Argentina |
| Lr09_JUJ_ARG | Landrace | Argentina |
| Lr10_JUJ_ARG | Landrace | Argentina |
| Lr11_MIS_ARG | Landrace | Argentina |
| Lr12_MIS_ARG | Landrace | Argentina |
| Lr13_MIS_ARG | Landrace | Argentina |
| Lr14_MIS_ARG | Landrace | Argentina |
| Lr15_MIS_ARG | Landrace | Argentina |
| Lr16_MIS_ARG | Landrace | Argentina |
| Lr17_MIS_ARG | Landrace | Argentina |
| Lr18_MIS_ARG | Landrace | Argentina |
| Lr19_MIS_ARG | Landrace | Argentina |
| Lr20_TUC_ARG | Landrace | Argentina |
| Lr21_TUC_ARG | Landrace | Argentina |
| Lr22_TUC_ARG | Landrace | Argentina |
| Lr23_TUC_ARG | Landrace | Argentina |
| Lr24_TUC_ARG | Landrace | Argentina |
| Lr25_TUC_ARG | Landrace | Argentina |
| Lr26_TUC_ARG | Landrace | Argentina |
| Lr27_TUC_ARG | Landrace | Argentina |
| Lr28_TUC_ARG | Landrace | Argentina |
| Lr29_TUC_ARG | Landrace | Argentina |
| Lr30_SRE_CUB | Landrace | Cuba |
| Lr31_SRE_CUB | Landrace | Cuba |
|  |  |  |

^a^ Identification

^b^ Cultivars refer to sold genotypes as seed lots through commercial providers

^c^ Country of the provider or origin of the landrace; Landraces from Cuba were provided by the New York Botanical Garden Herbarium, catalog number 1687090 et 1687091, collection number 5353, collected in Cuba in 1927 and 1931.

^d “^Eirete” type

^e “^Morita III” type

^f^ Genotype “D” from EUSTAS collection (Hastoy *et al*., 2019)

^g^ Genotype “C” from EUSTAS collection (Hastoy *et al*., 2019)

^h^ Genotype “Gawi” from EUSTAS collection (Hastoy *et al*., 2019)

^i “^Criolla” type

^j^ described in Moreno *et al*., 2016

Table S4: Distribution of the studied genotypes in the different clusters and admix following the analysis by Structure

| Genotype ID | Cultivar number | Cluster |
| --- | --- | --- |
| Cult05_CAN | Cultivar 2 | Cluster1 |
| Cult06_CAN | Cultivar 2 | Cluster1 |
| Cult07_CAN | Cultivar 2 | Cluster1 |
| Cult08_CAN | Cultivar 2 | Cluster1 |
| Cult09_CAN | Cultivar 2 | Cluster1 |
| Cult10_CAN | Cultivar 3 | Cluster1 |
| Cult12_CAN | Cultivar 3 | Cluster1 |
| Cult13_CAN | Cultivar 3 | Cluster1 |
| Cult70_GER | Cultivar 4 | Cluster1 |
| Cult71_GER | Cultivar 4 | Cluster1 |
| Cult72_GER | Cultivar 4 | Cluster1 |
| Cult73_GER | Cultivar 4 | Cluster1 |
| Cult74_GER | Cultivar 4 | Cluster1 |
| Cult76_GER | Cultivar 6 | Cluster1 |
| Cult78_GER | Cultivar 8 | Cluster1 |
| Cult79_GER | Cultivar 8 | Cluster1 |
| Cult80_GER | Cultivar 8 | Cluster1 |
| Cult81_GER | Cultivar 8 | Cluster1 |
| Cult82_GER | Cultivar 8 | Cluster1 |
| Cult83_ISR | Cultivar 1 | Cluster1 |
| Cult84_ISR | Cultivar 1 | Cluster1 |
| Cult85_ISR | Cultivar 1 | Cluster1 |
| Cult86_ISR | Cultivar 1 | Cluster1 |
| Cult87_ISR | Cultivar 1 | Cluster1 |
| Cult30_FRA | Cultivar 2 | Cluster2 |
| Cult32_FRA | Cultivar 4 | Cluster2 |
| Cult35_FRA | Cultivar 5 | Cluster2 |
| Cult37_FRA | Cultivar 7 | Cluster2 |
| Cult51_FRA | Cultivar 10 | Cluster2 |
| Cult52_FRA | Cultivar 10 | Cluster2 |
| Cult53_FRA | Cultivar 10 | Cluster2 |
| Cult54_FRA | Cultivar 10 | Cluster2 |
| Cult55_FRA | Cultivar 10 | Cluster2 |
| Cult56_FRA | Cultivar 10 | Cluster2 |
| Cult57_FRA | Cultivar 10 | Cluster2 |
| Cult58_FRA | Cultivar 11 | Cluster2 |
| Cult59_FRA | Cultivar 12 | Cluster2 |
| Cult60_FRA | Cultivar 12 | Cluster2 |
| Cult61_FRA | Cultivar 12 | Cluster2 |
| Cult62_FRA | Cultivar 12 | Cluster2 |
| Cult63_GER | Cultivar 1 | Cluster2 |
| Cult75_GER | Cultivar 5 | Cluster2 |
| Lr01_FOR_ARG | Landrace | Cluster2 |
| Lr02_JUJ_ARG | Landrace | Cluster2 |
| Lr04_JUJ_ARG | Landrace | Cluster2 |
| Lr05_JUJ_ARG | Landrace | Cluster2 |
| Lr06_JUJ_ARG | Landrace | Cluster2 |
| Lr07_JUJ_ARG | Landrace | Cluster2 |
| Lr08_JUJ_ARG | Landrace | Cluster2 |
| Lr09_JUJ_ARG | Landrace | Cluster2 |
| Lr10_JUJ_ARG | Landrace | Cluster2 |
| Lr11_MIS_ARG | Landrace | Cluster2 |
| Lr12_MIS_ARG | Landrace | Cluster2 |
| Lr13_MIS_ARG | Landrace | Cluster2 |
| Lr14_MIS_ARG | Landrace | Cluster2 |
| Lr15_MIS_ARG | Landrace | Cluster2 |
| Lr16_MIS_ARG | Landrace | Cluster2 |
| Lr17_MIS_ARG | Landrace | Cluster2 |
| Lr18_MIS_ARG | Landrace | Cluster2 |
| Lr19_MIS_ARG | Landrace | Cluster2 |
| Lr20_TUC_ARG | Landrace | Cluster2 |
| Lr21_TUC_ARG | Landrace | Cluster2 |
| Lr22_TUC_ARG | Landrace | Cluster2 |
| Lr23_TUC_ARG | Landrace | Cluster2 |
| Lr24_TUC_ARG | Landrace | Cluster2 |
| Lr25_TUC_ARG | Landrace | Cluster2 |
| Lr26_TUC_ARG | Landrace | Cluster2 |
| Lr27_TUC_ARG | Landrace | Cluster2 |
| Lr28_TUC_ARG | Landrace | Cluster2 |
| Lr29_TUC_ARG | Landrace | Cluster2 |
| Lr30_SRE_CUB | Landrace | Cluster2 |
| Lr31_SRE_CUB | Landrace | Cluster2 |
| Cult02_CAN | Cultivar 1 | Cluster3 |
| Cult100_SPA | Cultivar 1 | Cluster3 |
| Cult101_SPA | Cultivar 1 | Cluster3 |
| Cult102_SPA | Cultivar 2 | Cluster3 |
| Cult103_SPA | Cultivar 2 | Cluster3 |
| Cult104_SPA | Cultivar 2 | Cluster3 |
| Cult105_SPA | Cultivar 3 | Cluster3 |
| Cult106_SPA | Cultivar 3 | Cluster3 |
| Cult107_SPA | Cultivar 3 | Cluster3 |
| Cult108_SPA | Cultivar 3 | Cluster3 |
| Cult109_SPA | Cultivar 3 | Cluster3 |
| Cult11_CAN | Cultivar 3 | Cluster3 |
| Cult110_SPA | Cultivar 3 | Cluster3 |
| Cult111_SPA | Cultivar 3 | Cluster3 |
| Cult112_SPA | Cultivar 3 | Cluster3 |
| Cult113_SPA | Cultivar 3 | Cluster3 |
| Cult114_SPA | Cultivar 3 | Cluster3 |
| Cult29_FRA | Cultivar 1 | Cluster3 |
| Cult31_FRA | Cultivar 3 | Cluster3 |
| Cult33_FRA | Cultivar 5 | Cluster3 |
| Cult34_FRA | Cultivar 5 | Cluster3 |
| Cult36_FRA | Cultivar 6 | Cluster3 |
| Cult38_FRA | Cultivar 5 | Cluster3 |
| Cult39_FRA | Cultivar 5 | Cluster3 |
| Cult40_FRA | Cultivar 5 | Cluster3 |
| Cult41_FRA | Cultivar 5 | Cluster3 |
| Cult42_FRA | Cultivar 8 | Cluster3 |
| Cult43_FRA | Cultivar 5 | Cluster3 |
| Cult44_FRA | Cultivar 5 | Cluster3 |
| Cult45_FRA | Cultivar 5 | Cluster3 |
| Cult46_FRA | Cultivar 9 | Cluster3 |
| Cult47_FRA | Cultivar 9 | Cluster3 |
| Cult48_FRA | Cultivar 9 | Cluster3 |
| Cult49_FRA | Cultivar 9 | Cluster3 |
| Cult50_FRA | Cultivar 9 | Cluster3 |
| Cult64_GER | Cultivar 2 | Cluster3 |
| Cult65_GER | Cultivar 3 | Cluster3 |
| Cult66_GER | Cultivar 3 | Cluster3 |
| Cult67_GER | Cultivar 3 | Cluster3 |
| Cult68_GER | Cultivar 3 | Cluster3 |
| Cult69_GER | Cultivar 3 | Cluster3 |
| Cult77_GER | Cultivar 7 | Cluster3 |
| Cult88_NET | Cultivar 1 | Cluster3 |
| Cult89_NET | Cultivar 1 | Cluster3 |
| Cult90_NET | Cultivar 1 | Cluster3 |
| Cult91_NET | Cultivar 1 | Cluster3 |
| Cult92_NET | Cultivar 1 | Cluster3 |
| Cult93_NET | Cultivar 2 | Cluster3 |
| Cult94_NET | Cultivar 2 | Cluster3 |
| Cult95_NET | Cultivar 2 | Cluster3 |
| Cult96_NET | Cultivar 2 | Cluster3 |
| Cult97_SPA | Cultivar 1 | Cluster3 |
| Cult98_SPA | Cultivar 1 | Cluster3 |
| Cult99_SPA | Cultivar 1 | Cluster3 |
| Cult01_CAN | Cultivar 1 | Admix |
| Cult03_CAN | Cultivar 1 | Admix |
| Cult04_CAN | Cultivar 1 | Admix |
| Cult14_CHI | Cultivar 1 | Admix |
| Cult15_CHI | Cultivar 1 | Admix |
| Cult16_CHI | Cultivar 1 | Admix |
| Cult17_CHI | Cultivar 1 | Admix |
| Cult18_CHI | Cultivar 1 | Admix |
| Cult19_CHI | Cultivar 1 | Admix |
| Cult20_CHI | Cultivar 1 | Admix |
| Cult21_CHI | Cultivar 1 | Admix |
| Cult22_CHI | Cultivar 2 | Admix |
| Cult23_CHI | Cultivar 2 | Admix |
| Cult24_CHI | Cultivar 2 | Admix |
| Cult25_CHI | Cultivar 2 | Admix |
| Cult26_CHI | Cultivar 2 | Admix |
| Cult27_CHI | Cultivar 2 | Admix |
| Cult28_CHI | Cultivar 2 | Admix |
| Lr03_JUJ_ARG | Landrace | Admix |
